# Influence of Diet on Reproducible Corticosterone Levels in a Mouse Model of Maternal Separation with Early Weaning

**DOI:** 10.3390/life14070880

**Published:** 2024-07-15

**Authors:** Jamie Y. Choe, Michael Donkor, Roland J. Thorpe, Michael S. Allen, Nicole R. Phillips, Harlan P. Jones

**Affiliations:** 1 Texas College of Osteopathic Medicine, University of North Texas Health Science Center, Fort Worth, TX 76107, USA; jamiechoe@my.unthsc.edu; 2 Department of Microbiology, Immunology & Genetics, University of North Texas Health Science Center, Fort Wort, TX 76107, USA; michaeldonkor@my.unthsc.edu (M.D.); michael.allen@unthsc.edu (M.S.A.); nicole.phillips@unthsc.edu (N.R.P.); 3 Hopkins Center for Health Disparities Solutions, Johns Hopkins Bloomberg School of Public Health, Baltimore, MD 21205, USA; rthorpe@jhu.edu; 4 Institute for Translational Research, University of North Texas Health Science Center, Fort Worth, TX 76107, USA; 5 Institute for Health Disparities, University of North Texas Health Science Center, Fort Worth, TX 76107, USA

**Keywords:** animal housing, corticosterone, diet, early life stress, mouse, early wean

## Abstract

Maternal separation with early weaning (MSEW) is a popular early life stress (ELS) model in rodents, which emulates childhood neglect through scheduled mother-offspring separation. Although variations of ELS models, including maternal separation and MSEW, have been published for the mouse species, the reported results are inconsistent. Corticosterone is considered the main stress hormone involved in regulating stress responses in rodents—yet generating a robust and reproducible corticosterone response in mouse models of ELS has been elusive. Considering the current lack of standardization for MSEW protocols, these inconsistent results may be attributed to variations in model methodologies. Here, we compared the effects of select early wean diet sources—which are the non-milk diets used to complete early weaning in MSEW pups—on the immediate stress phenotype of C57BL/6J mice at postnatal day 21. Non-aversive handling was an integral component of our modified MSEW model. The evaluation of body weight and serum corticosterone revealed the early wean diet to be a key variable in the resulting stress phenotype. Interestingly, select non-milk diets facilitated a stress phenotype in which low body weight was accompanied by significant corticosterone elevation. Our data indicate that dietary considerations are critical in MSEW-based studies and provide insight into improving the reproducibility of key stress-associated outcomes as a function of this widely used ELS paradigm.

## 1. Introduction

Early life stress/adversity (ELS), often referred to in humans as adverse childhood experiences, is now widely recognized as harmful early life exposures/events that can have long-term consequences that present many years later in adulthood [1,2,3,4,5,6]. Epidemiological studies have identified significant associations between adverse childhood experiences and risk for pathology in adulthood [7,8,9,10,11,12]. Although ELS has arguably been studied the most extensively in the basic sciences in the context of psychiatric [13,14,15] and cardiometabolic disorders [8,16], an increasing number of disciplines have shown interest in studying ELS in recent years [17,18,19,20,21].

Animal models are valuable tools for preclinical studies. A strength of basic science research is the ability to conduct controlled experiments with defined variables that allow researchers to determine causality. This focus on unraveling causal mechanisms behind ELS-mediated outcomes has gained attention across disciplines and is appreciable in the literature through the variety of published animal-based ELS studies [21,22,23,24,25,26]. However, research within the basic sciences is inherently limited by the quality and accessibility of animal models available for in vivo studies. ELS researchers have long been plagued by the lack of robust, reproducible animal models—namely in mice. Mice are model organisms of choice due to the high genetic similarity between mice and humans [27]. Other benefits of mouse models include the species’ favorable breeding capacity, sequenced (and manipulable) genome, and readily available species-compatible assays. Although improvements have been made over time to ELS animal models (e.g., maternal separation alone, early weaning alone, wire cage floors or limited bedding models, and combined maternal separation with early weaning), each model has limitations [28].

To date, the maternal separation with early weaning (MSEW) paradigm is one of the most popular models used to study ELS with the mouse species. The MSEW paradigm was first described in 2010 by George et al. [29] in response to alternate versions of ELS models, such as maternal separation or maternal deprivation, yielding mixed results in mice [30]. Although George et al. were unable to demonstrate significant corticosterone effects, their version of the MSEW model was validated based on assessing behavioral outcomes [29]. MSEW-based models of ELS are an attractive option based on their relatively low-cost design and accessibility (i.e., does not require special cage conditions) as well as widespread application within the C57BL/6J inbred mouse line.

A persistent issue observed with MSEW-based models is high variability in resulting corticosterone responses [29,31,32,33]. Furthermore, many publications utilizing MSEW report differences in their methods (e.g., daily hours of dam-pup separation, specific early wean age, decision to cull (artificial equalization of the number and/or sex distribution of offspring in a litter) or keep natural litters intact, and type of non-milk diet provided to the pups upon early weaning. An integral aspect of the MSEW paradigm involves early weaning pups to recapitulate and exacerbate an environment of childhood neglect (i.e., permanently separate the dam and pups before the standard wean date). This necessitates switching early-weaned pups (who no longer have access to the dam’s milk) to a non-milk diet source for sustenance. Published meta-analyses and reviews of the current literature discussing the heterogeneity between ELS studies [34,35] further highlight the need to explore the potential impact of diet as a variable in the early wean protocol, which, to date, has not been considered. Most published MSEW-based work reports the early wean diet as “standard chow”. However, the supplier/type of pellets considered “standard” can differ between academic institutions or even between housing units within a given institution’s animal facilities (e.g., conventional vs. barrier housing halls). Furthermore, the use of both rats and mice in MSEW studies combined with their contradictory results have contributed to the lack of consensus permeating the current literature [30,36].

When considering the model’s reputation for inconsistent corticosterone results, we believe a deeper dive into the potential implications of diet as a variable in the context of MSEW is warranted. Although models of ELS have historically been validated with behavioral studies [34,37], the need and pursuit of an improved MSEW model capable of generating consistent corticosterone responses should not be ignored. In this study, we compare the effects of four different non-milk diet sources when used as part of early weaning within the MSEW protocol for ELS in C57BL/6J mice. We hypothesize that diet is an essential variable in the context of the described MSEW paradigm to achieve reproducible corticosterone responses.

## 2. Materials and Methods

### 2.1. Animals

Male and female C57BL/6J mice aged 4–6 weeks old were ordered from Jackson Laboratory (Bar Harbor, ME, USA) to be used as breeders to produce the newborn pups used in this study. Breeder animals were acclimated to the animal facility for at least 1 week before starting in-house breeding. The female dams and stud males were randomly assigned to an experimental group (control or MSEW) and retained their assignment throughout the experiment and all subsequent breeding cycles. Animals were housed under a 12:12 h light/dark cycle (lights on at 7:00 a.m.) with constant temperature (20.6–23.3 °C) and 30–70% humidity in conventional mouse cages with overhead feeders and an auto-water lixit system (purified water treated with reverse osmosis). Breeder animals were housed with ad libitum access to food and water. Autoclaved acrylic tunnels were used to transfer and handle dams for the experimental protocol. Animals were habituated to transfer tunnels prior to the first round of breeding. Breeder females were housed with stud males (1:1 or 2:1 ratio) for a period of 2 weeks to ensure conception during the estrous period. At 2 weeks, breeder females were separated into individual cages and monitored for litters daily. The day of birth was defined as postnatal day 0 (PD0). Litters were not culled to represent the natural variation between size and sex distribution of a family unit (which we define as the dam and her pups). Pups were sexed at PD4–7 based on the observation of a pigmented spot in the anogenital region (procedure described in Wolterink-Donselaar et al. [38]). Pup sex was re-confirmed on PD21 at the time of sacrifice using anogenital distance. Early-weaned pups were group-housed with their natural male and female littermates during the PD14–21 window. Both MSEW dams and control dams were housed with the transfer tunnel for the entirety of PD0–21. All animal procedures were performed in accordance with the rules and regulations of the Institutional Animal Care Use Committee (IACUC) at the University of North Texas Health Science Center.

### 2.2. MSEW Protocol

**Figure 1:**
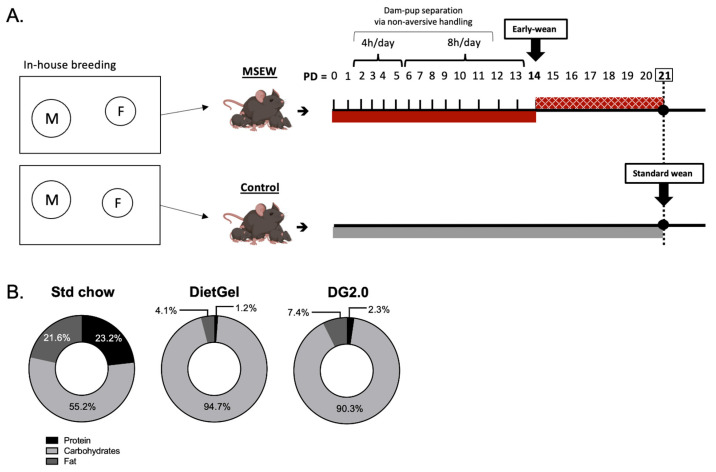
Overview of the maternal separation with early weaning (MSEW) model of ELS. (**A**) Schematic of the MSEW model compared to control conditions. In-house breeding between a stud male (“M”) and breeder female (“F”) produced the newborn pups for experiments. Postnatal day (PD) indicates litter age in days at the respective time points of the MSEW protocol with crosshatching used to represent the early wean window (pups switched to non-milk diet). PD0 is defined by day of birth. (**B**) Macronutrient profiles of the non-milk diets: moist PicoLab^®^ 5058 pellets (Std chow), Clear H_2_O^®^ DietGel Recovery (DietGel), and the manufacturer’s reformulated DietGel Recovery (DG2.0).

The maternal separation started on PD2 by removing the dam from her pups in the home cage using non-aversive handling into a clean cage with ad libitum access to food and water. Figure 1A shows the separation schedule utilized in this study with dam-pup separation lasting for 4 h during PDs 2–5 and 8 h during PDs 6–13. During the separation period, pups remained together with littermates in the home cage, which was well-ventilated and kept over a heating pad to assist with thermoregulation by maintaining a constant cage temperature (32–34 °C). Dams had ad libitum access to food and water. The dam and pups were kept in separate rooms to prevent vocalization effects. At the end of the separation period, the dam was returned to the home cage with her pups. The tunnel transfer method is a non-aversive handling technique proven to reduce unintended stress effects associated with conventional tail handling [39,40,41]. The tunnel transfer method was used exclusively to handle mice during the scheduled dam-pup separation portion of our MSEW protocol (Figure 1A). Early weaning occurred on PD14 by relocating all pups of a given litter into a clean cage, away from the dam. MSEW pups of a given litter were kept together during the early wean window (PDs 14–21) and fed one of the following non-milk diets: (1) PicoLab^®^ 5058 pellets (Standard (Std) chow) moistened with reverse-osmosis-purified tap water, (2) Clear H_2_O^®^ DietGel^®^ Recovery (DietGel), (3) a combination of DietGel (1 gel cup) with standard chow (18–19 g), or the (4) reformulated Clear H_2_O^®^ DietGel^®^ Recovery (DG2.0). During this study, the manufacturer of DietGel^®^ released a reformulated version of DietGel^®^ Recovery to replace the original version of this product. To distinguish between these two formulations of the DietGel^®^ Recovery product, we refer to them here as DietGel (original formulation of DietGel^®^ Recovery; no longer available for purchase) and DG2.0 (reformulated DietGel^®^ Recovery; at the time of manuscript submission, this product formulation is available for purchase on the Clear H_2_O^®^ manufacturer website). The non-milk diets utilized in this study were selected based on the widespread use of Clear H_2_O^®^ products to support animals in research, DLAM staff recommendations, and the type of “standard chow” used in our animal housing facility (PicoLab^®^ 5058). Macronutrient information for the non-milk diets included in this study was obtained from the respective manufacturer and is displayed as percentages for macronutrient comparison in Figure 1B. Non-milk diets were monitored and refreshed every 2–3 days. Litters sized *n* = 1–4 received one DietGel or DG2.0 cup every 2–3 days while litters *n* > 4 received two DietGel or DG2.0 cups every 2–3 days. Old gel remnants were removed from the cage prior to replenishing with fresh DietGel or DG2.0. The combination of DietGel with standard chow (DietGel + chow) was prepared by inserting standard chow pellets into the DietGel gel cup formulation, thereby reducing spatial cognition or discriminatory selection between the diet sources. Control pups were naturally reared by dams under the described animal housing conditions and weaned on the PD21 standard wean date. Animals were euthanized at PD21 for tissue collection. The body weights of all pups were assessed on PD21.

### 2.3. Tissue Collection and Preparation

Mice were euthanized at PD21 via carbon dioxide (CO_2_) inhalation and a secondary method of exsanguination (cardiac puncture). Blood was collected (at a time of day between 7:30 a.m.–12:00 p.m.) by terminal cardiac puncture and allowed to clot at room temperature, followed by centrifugation at 10,000× *g* for 10 min at 4 °C. Serum samples were collected as the supernatant and stored at −20 °C until analysis.

### 2.4. Corticosterone ELISA

Blood serum corticosterone levels were measured using a validated commercial corticosterone ELISA kit (EIACORT, Invitrogen™, Waltham, MA, USA) according to the manufacturer’s instructions. Standards were run in duplicate with assay controls per the manufacturer’s instructions. Each sample represented one animal (one sample per animal), and assays were run on single detection.

### 2.5. Statistical Analyses

All figures and analyses were completed using GraphPad Prism 9 software. Statistical analyses were performed as described in figure legends using an alpha-level criterion of 0.05 for statistical significance. Results and error bars represent mean ± SD with *** <0.001, ** <0.01, * <0.05. Two-way ANOVA was used to analyze the effects of early wean diet, sex, and their interaction (two-factor analysis). Significant two-way ANOVAs were followed by Tukey’s post-hoc multiple comparisons to detect differences between individual groups. The Kruskal–Wallis test followed by Dunn’s post-hoc was used to analyze the effect of an early wean diet on the dependent variable (one-factor analysis). Pilot studies revealed significant inter-animal variation in the serum corticosterone data within experimental groups. Tukey’s box-plot method (1.5 times interquartile range (IQR)) was used to flag the presence of potential outliers in the raw dataset. Grubb’s test was used to detect and identify the outliers for removal. Outliers were removed from the corticosterone dataset prior to generating figures and two-way ANOVAs (a total of three outliers were identified and removed from groups DietGel male, DietGel female, and DietGel + chow male). The normal distribution of data was assessed using tests of normality and lognormality in GraphPad Prism 9 software. Where applicable, the Kruskall–Wallis one-way ANOVA was utilized as a non-parametric analysis of variance. The nonparametric Spearman correlation was used to examine the relationship between weight and corticosterone at a 95% confidence interval and two-tailed *p*-value (see Table S1 for complete Spearman rank correlation test statistics).

## 3. Results

### 3.1. Variation in the Non-Milk Diet Alone Impacts Body Weight

**Figure 2:**
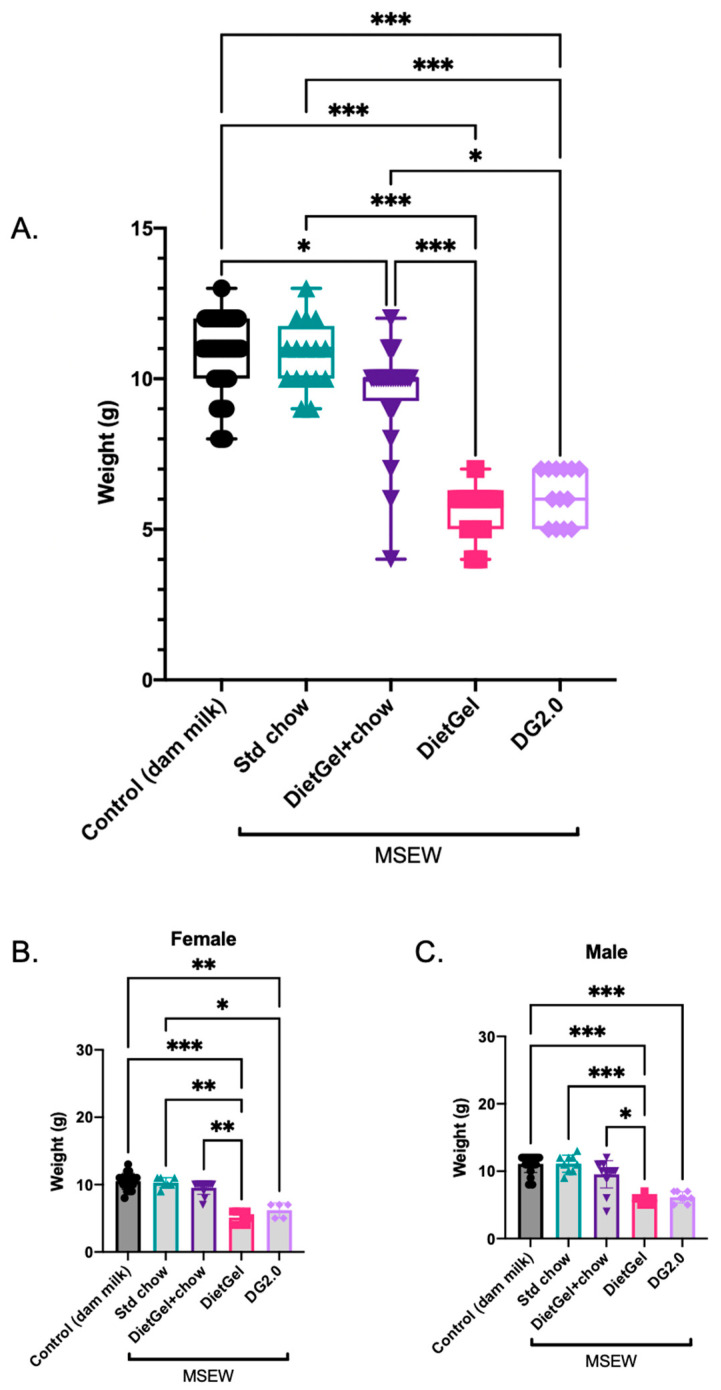
Body weight comparisons of mice based on PD14–21 diet for (**A**) combined sexes (Control *n* = 42; MSEW DietGel *n* = 24; MSEW Std chow *n* = 16; MSEW DietGel + chow *n* = 28; MSEW DG2.0 *n* = 13). Dam milk is the control diet. Box extends from 25th to 75th percentiles. Line in the middle represents the median. Whiskers extend to minimum and maximum values. Sex-based body weight comparisons for (**B**) females (Control *n* = 20, MSEW DietGel *n* = 9, MSEW Std chow *n* = 7, MSEW DietGel + chow *n* = 13, MSEW DG2.0 *n* = 5), and (**C**) males (Control *n* = 22, MSEW DietGel *n* = 15, MSEW Std chow *n* = 9, MSEW DietGel + chow *n* = 15, MSEW DG2.0 *n* = 8). Mice aged PD21. Error bars on bar graphs represent mean ± SD. Asterisks denote significance according to Kruskal–Wallis test (**A**–**C**) with Dunn’s post-hoc (**A**) at *p* < 0.001 (***), *p* < 0.01 (**), *p* < 0.05 (*).

To understand if the non-milk diet source used for early weaning has any influence on the physical characteristics of MSEW pups, we compared the effect of the early wean window diet (PD14–21) on body weight while holding all other experimental conditions for MSEW constant. Mean body weight data for the control group and four MSEW subgroups are shown in Figure 2A. The type of non-milk diet used during early weaning had a significant impact on the body weight of the MSEW pups at age PD21, with significant variations found within MSEW subgroups. Notably, the DietGel and DG2.0 diets produced the lowest body weights and were significantly lower compared to both control and standard chow-fed mice. Data for female (Figure 2B) and male (Figure 2C) body weights are combined in Figure 2A based on preliminary studies showing no significant differences in body weight between sexes (see Table 1 for complete Kruskal–Wallis test statistics).

### 3.2. Select Non-Milk Diets Increased Circulating Corticosterone

**Figure 3:**
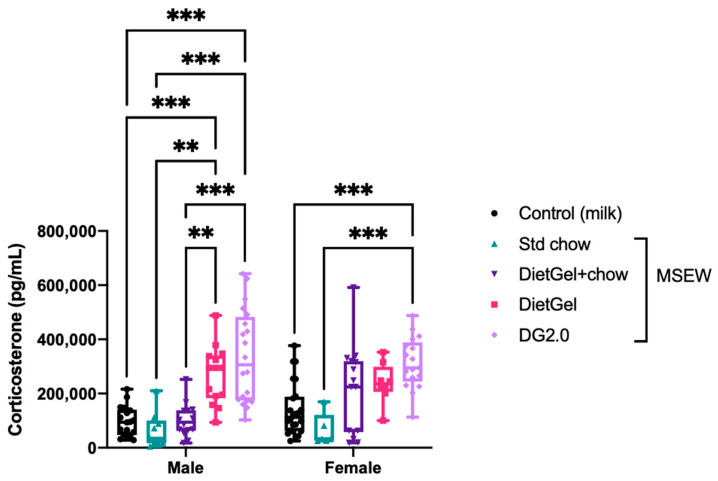
Serum corticosterone measured at PD21 (Control: *n* = 18 male, *n* = 24 female; MSEW DietGel: *n* = 14 male, *n* = 8 female; MSEW Std chow: *n* = 9 male, *n* = 7 female; MSEW DietGel + chow: *n* = 14 male, *n* = 15 female; MSEW DG2.0: *n* = 20 male, *n* = 17 female). Outliers were identified in the corticosterone raw dataset with Grubb’s test and removed prior to generating figures and applying the indicated tests to assess statistical significance. Error bars represent mean ± SD. Asterisks denote significance according to two-way ANOVA with Tukey’s post-hoc at *p* < 0.001 (***), *p* < 0.01 (**).

**Figure 4:**
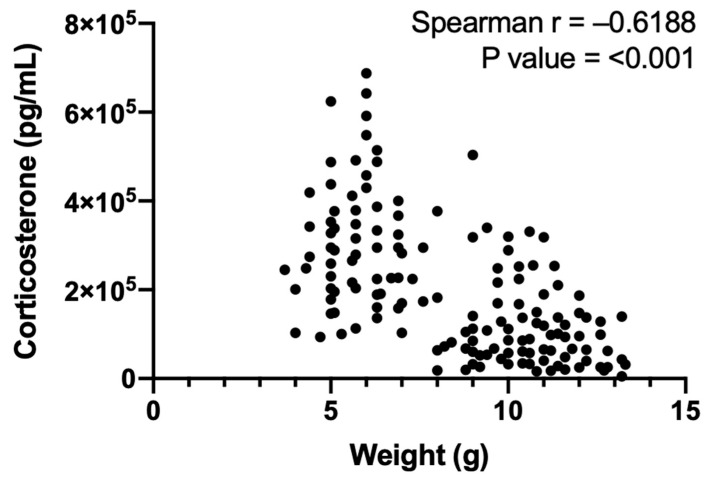
Body weight is correlated to serum corticosterone levels in PD21 mice. Spearman rank correlation of all mice included in the study (*n* = 147 total).

Corticosterone levels varied significantly between MSEW pups based on the type of early wean diet (Figure 3; Table 2). Altering the non-milk diet alone revealed a highly variable peripheral corticosterone response. Compared to male controls, corticosterone was significantly elevated in male MSEW DietGel pups. Significant elevations in corticosterone were observed in both male and female MSEW DG2.0 compared to sex-matched controls. Our data show no sex differences between groups and no difference between the peripheral corticosterone levels in standard chow-fed MSEW pups compared to controls. Furthermore, Spearman rank revealed a correlation between body weight changes and corticosterone levels (Figure 4; see Table S1 for Spearman rank correlation test statistics).

## 4. Discussion

The MSEW paradigm is notorious for its inconsistent effects on corticosterone in mice. This model induces stress in pups as a function of tactile maternal deprivation and neglect based on the scheduled dam–pup separation and permanent early weaning. We have shown that peripheral corticosterone is impacted by the type of non-milk diet used for early-weaned pups as part of the widely used MSEW model. We are unaware of any other study that has looked at the role of diet in the MSEW model and its impact on the resulting corticosterone response. Our study provides evidence for dietary considerations in the MSEW mouse model based on changes in body weight and serum corticosterone. Although not currently standardized, it is common practice to use moist standard chow to early wean mice in MSEW studies [29,32,36]. By isolating the non-milk diet in our study, our data reveal the early wean diet to be a critical variable in designing MSEW studies. Based on historical challenges for the MSEW model to generate robustly elevated corticosterone levels in mice, the results of this study may provide insight into identifying an optimal early wean diet to achieve the desired MSEW stress phenotype (e.g., significantly elevated corticosterone). Our data showed no difference between the peripheral corticosterone levels in standard chow-fed MSEW pups compared to controls, which is consistent with the trends reported in the original publication characterizing the MSEW model [29].

To our knowledge, we are also the first to utilize the tunnel transfer method of non-aversive handling as an integral part of the maternal separation component of MSEW (which necessitates handling the dam daily). Tail-handling (i.e., lifting the mouse by the base of its tail by taking it between the handler’s thumb and forefinger) is considered standard in laboratory research and is therefore presumed to be the method of handling used by other groups performing MSEW unless otherwise stated or specified. Considering mice are natural prey animals, frequent tail-handling is known to elicit independent stress effects by inducing aversion and anxiety in mice [42,43]. Stress effects in ELS studies may therefore be confounded based on the method of handling used.

In this study, the two groups with the greatest elevations in corticosterone (MSEW DietGel and MSEW DG2.0) also had the lowest body weights. These results indicate that low body weight may be favorable in the present model to facilitate peripheral corticosterone elevation in mice intended to model conditions of ELS. Low body weight may therefore reflect an increased level of stress in the context of the ELS environment (emulated by MSEW), although further studies are required to confirm this association beyond the correlation analysis provided here. It is also possible that MSEW pups are inherently subject to weight loss following early weaning due to an initial aversion to the novel diet (i.e., switching from dam’s milk to the novel non-milk diet at PD14 as part of early weaning). However, the standard chow-fed pups interestingly experienced no change in body weight compared to controls, making it less likely for the observed weight loss to be attributed to the novelty of the non-milk diet alone. This suggests that the type of non-milk diet itself, rather than novel food aversion, is responsible for the effects of early weaning on body weight. Notably, direct effects on the adrenal glands were not assessed in the present study. Evaluating adrenal gland alterations (e.g., measuring adrenal gland weights in the context of body weight changes) would provide additional insight. For example, it is possible that adrenal glands undergo hypertrophy in connection with increased corticosterone responses [44]. Further investigation is required to ascertain these effects, which would enhance our understanding of dietary effects on the resulting corticosterone-based stress phenotype.

As with any animal model-based experiment, this study comes with limitations that should be considered in the interpretation of results. First, the findings of this study are limited to the PD21 time point. Although we anticipate later time points to reflect similar trends in differences between control and MSEW animals, additional studies are required to determine if, and for how long, these trends persist. Because PD21 is the final day of the MSEW protocol (Figure 1A), observations at the PD21 timepoint may be considered the immediate results of ELS exposure. Since many researchers are interested in the long-term effects of ELS exposure, follow-up studies to evaluate these biological endpoints at set intervals post-MSEW protocol completion would be valuable to provide insight into the longevity of the described ELS-related phenotype (e.g., Are corticosterone elevations still observed at later timepoints? Does the body size of MSEW mice recover to resemble the controls?). Second, the two DietGel^®^ Recovery formulations included in this study (DietGel and DG2.0) lack a vitamin matrix. DietGel^®^ Recovery is a purified water gel containing 60% water and fortified with calories and minerals (providing 155 kcals/100 g per packaged cup). It is known that nutrition plays an important role in early life, and malnutrition itself represents a form of stress [45,46]. Therefore, it is possible that vitamin deficiencies may account for the observed impacts of DietGel and DG2.0. Further evaluation of our version of the MSEW protocol, as reported here, should be pursued with other formulations of DietGel^®^ offered by the manufacturer that include the vitamin matrix. We also acknowledge that the non-milk diets tested here have macronutrient differences (Figure 1B). Yet, the objective and emphasis of the present study is on the ability of the diet variable to significantly contribute to the visualized changes in corticosterone as part of the MSEW paradigm—which has not been shown or investigated by others. The non-milk diets were selected based on their common use in research facilities to support laboratory rodents. Here, we tested a range of macronutrient-content diets (i.e., high-carbohydrate/low-protein/low-fat DietGels as compared to the more balanced macronutrient profile of Std chow) and have shown the impact these macronutrient differences can have on corticosterone at PD21 as part of MSEW.

## 5. Conclusions

Adding to the existing literature, we have shown evidence in support of moving towards a standardized version of the MSEW model based on early wean (non-milk) diet variation leading to significantly varied outcomes on corticosterone. Our data show that nuances in the MSEW protocol—such as the early wean diet alone—can impact key biological outcomes that comprise the stress phenotype (i.e., peripheral corticosterone). Based on our findings, we believe that diet should be considered a key variable in the MSEW study design and encourage non-aversive handling methods to improve the consistency of applied exogenous stressors between control and MSEW mice. While we expect MSEW to continue growing in popularity and encourage other researchers to utilize this model, our data suggest that standardization may be critical to support the reproducibility and reliability of MSEW-based studies moving forward.

## Figures and Tables

**Table 1:** Kruskal–Wallis test statistics for body weight comparisons at PD21 based on PD14–21 diet for (**A**) combined sexes, (**B**) females, and (**C**) males. Asterisks denote significance at *p* < 0.001 (***).

Kruskal-Wallis Test (Multiple Comparisons)	
**(A) Combined sexes**	
*p* value	<0.001
Exact or approximate *p* value?	Approximate
*p* value summary	***
Do the medians vary signif. (*p* < 0.05)	Yes
Number of groups	5
Kruskal-Wallis statistic	82.58
**(B) Females**	
*p* value	<0.001
Exact or approximate *p* value?	Approximate
*p* value summary	***
Do the medians vary signif. (*p* < 0.05)	Yes
Number of groups	5
Kruskal-Wallis statistic	35.38
**(C) Males**	
*p* value	<0.001
Exact or approximate *p* value?	Approximate
*p* value summary	***
Do the medians vary signif. (*p* < 0.05)	Yes
Number of groups	5
Kruskal-Wallis statistic	46.98

**Table 2:** Two-way ANOVA statistics for serum corticosterone measured at PD21.

ANOVA Table	F (DFn, DFd)	*p* Value
**Interaction**	F (4, 136) = 1.874	*p* = 0.12
**Sex**	F (1, 136) = 1.095	*p* = 0.30
**Treatment**	F (4, 136) = 25.38	*p* < 0.001

## Data Availability

The data that support the findings of this study are available from the corresponding author upon reasonable request.

## Supplementary Materials

The following supporting information can be downloaded at: https://www.mdpi.com/article/10.3390/life14070880/s1, Table S1: Spearman rank correlation test statistics. Video S1: Demonstration of the tunnel transfer method for non-aversive handling used to perform daily dam–pup separation sessions as part of the MSEW protocol on PDs 2–13.
