# Supplementary material for: Influence of Diet on Reproducible Corticosterone Levels in a Mouse Model of Maternal Separation with Early Weaning
Source: Life (Basel). 2024 Jul 15;14(7):880. doi: 10.3390/life14070880 (PMC11277828; doi:10.3390/life14070880)
Supplement: Supplementary file 1 [file life-14-00880-s001.zip › life-3067782-Table S1.pdf]

Table S1. Spearman rank correlation test statistics.

Spearman	
r	-0.6188
95% confidence interval	-0.7120 to -0.5042
P value (two-tailed)	<0.001
P value summary	***
Exact or approximate P value?	Approximate
Significant? (alpha = 0.05)	Yes
